# Modern Sociology

**Published:** 1901-10-05

**Authors:** 


### Modern Sociology.

### GARDEN CITIES.

The tendency of modern civilisation is for people to aggregate together in masses, and this applies both to workers and drones, the former being attracted by business and the latter by pleasure. But if the social centres of gravity exist in the capitals and large towns, and if many of the leisured classes choose to live in a series of dark cupboards at a rent which would command a country mansion, that does not alter the fact that the people who are compelled to work in factories should be surrounded by as much fresh air and sunshine as possible. One manufactory employing hundreds of workers necessarily attracts other subsidiary businesses to cater to their wants and it is therefore impossible to isolate each factory and plant it in the open fields. Again, in spite of cheap working men's trains, trams, electric tubes, and omnibuses it is found impossible to induce many to leave the towns and live far from their work. The problem is, therefore, to combine a working town and a rural district maintaining, as far as possible, its natural rusticity. This the Garden City Association has set itself to solve, and the practical outcome is the model village of Bournville, founded by Messrs. Cadbury and ceded to a body of trustees for the people. The scheme is based on sound economic principles, the idea being to acquire land at its agricultural price, to build a garden city upon it, and to retain for the benefit of the community the increased value of the land which would accrue. In other words, the "unearned increment," instead of finding its way into the coffers of the ground landlords, would belong to the inhabitants, the original shareholders being restricted to 4 or 5 per cent. interest on their capital.

The idea of the garden city is to allow five persons to the acre, and it is estimated by Mr. Ralph Neville, K.C., that on this scale the counties of Surrey, Kent, Sussex, Hampshire and Berkshire, would absorb the population of London nearly four times over, calculating on the 1891 census.

The plan as enunciated by Mr. Ebenezer Howard is to acquire, say, 6,000 acres of land and to build a town for a population of 30,000 in the centre which would occupy about 1,000 acres and be surrounded by an agricultural area five times as large. From a public health point of view the scheme possesses the greatest advantages, and the conception of a modern town with the latest sanitary principles put into practice, with wide open streets, with parks and gardens and with no "slums" appears almost Utopian. There are undoubtedly difficulties in the way, but they are by no means insurmountable. The success or failure of such schemes will depend chiefly on the manufacturers, but there is no reason why, with railway facilities, such industries as the textile, bootmaking, pottery and lace should not be carried on as successfully in garden cities as at present. Iron, coal, and other metal works must be carried on near the spot where nature has placed the raw products, and it is manifestly impossible to maintain agricultural surroundings close to such industries. Presumably these workers must still continue to respire an atmosphere heavily charged with carbon and sulphur, until some method is discovered of perfecting the combustion of coal.

Perhaps in time we may find that instead of factory towns being denoted by factory chimneys the power to drive machinery will be distributed by electricity, and when that time arrives the possibilities before the Garden City will be vastly widened. There are many who, looking at manufacturing towns as they now exist, would shudder at the thought of defiling the rural districts by planting mills out in the clean country. With the electric distribution of power, however, all this would be changed: the mill would come, but the smoke would be left behind, and the city might be a garden even though it might be clustered round a group of factories.

In the model village of Bourneville the factories are to be worked by electricity produced by the Mond gas system, so that there will be no chimneys and no smoke to spoil the gardens. If this be practicable the life of the community will indeed be idyllic. Sunlight, fresh air, green fields and multi-coloured flowers will take the place of dismal fogs, stunted smoke-begrimed patches of so-called grass and hard paved courts and alleys. With such surroundings the physical, mental, and moral health of the community could not fail to be greatly improved. The stunted, narrow-chested, unfertile denizen of our great towns would become a well-developed, strong and happy father of a healthy and prosperous family. Instead of prowling the pavements or spending his evenings in the public-house, he would be occupied in his garden or in enjoying a country walk near at hand. Instead of the ceaseless hum of traffic he would hear the song of nature, and instead of starting to his work in the morning with a heavy, dull and listless feeling he would appear fresh and bright. His physical condition would improve and probably he would soon learn that he was not a hireling cog in the great social machine but an integral unit benefiting by the improvements which he had helped to create.

We must also hope that the present idea that it is the duty of the worker to limit his output to a certain amount in order that the quantity of necessary work should be spread over the greatest number of labourers would be replaced by a more accurate knowledge of the elementary principles governing co-ordinated work. Undoubtedly the rising generation would benefit in every way. For growing children country surroundings are absolutely essential if we wish them to develop into strong healthy men and women. After their school hours, cricket and other games in the open parks would take the place of the amusements of the streets. Again, agriculture would be stimulated by the fact that a market existed close at hand and the produce would not be spoilt by a long journey by road or rail. Whatever may be the result and whether it be practical permanently to restrict the area to be covered with buildings, as is suggested, there can be no doubt that the scheme in itself deserves every encouragement and that the benefit to the workers themselves and consequently to the quality of their work would be very great if carried on under such circumstances.

